# PRIMMO study protocol: a phase II study combining PD-1 blockade, radiation and immunomodulation to tackle cervical and uterine cancer

**DOI:** 10.1186/s12885-019-5676-3

**Published:** 2019-05-28

**Authors:** Sandra Tuyaerts, An M. T. Van Nuffel, Eline Naert, Peter A. Van Dam, Peter Vuylsteke, Alex De Caluwé, Sandrine Aspeslagh, Piet Dirix, Lien Lippens, Emiel De Jaeghere, Frédéric Amant, Katrien Vandecasteele, Hannelore Denys

**Affiliations:** 1 Division of Gynecologic Oncology, Department of Oncology, KU Leuven, Leuven, Belgium; 2 Leuven Cancer Institute (LKI), Leuven, Belgium; 3 Anticancer Fund, Strombeek-Bever, Belgium; 4 Division of Medical Oncology, UZ Gent, Ghent, Belgium; 5 Cancer Research Institute Gent (CRIG), Ghent, Belgium; 6 Division of Gynecologic Oncology and Senology, University Hospital Antwerp, Antwerp, Belgium; 7 Division of Oncology, CHU UCL Namur, Sainte Elisabeth, Namur, Belgium; 8 Division of Radiation Oncology, Institut Jules Bordet, Brussels, Belgium; 9 Division of Radiation Oncology, Iridium Cancer Network, Antwerp, Belgium; 10 Division of Molecular Imaging, Pathology, Radiotherapy & Oncology (MIPRO), University of Antwerp, Antwerp, Belgium; 11 Division of Gynecology & Obstetrics, UZ Leuven, Leuven, Belgium; 12 Center for Gynecologic Oncology Amsterdam (CGOA), Amsterdam, the Netherlands; 13 Division of Radiation Oncology, UZ Gent, Ghent, Belgium

**Keywords:** PD-1 blockade, Radiation, Immune modulation, Tumor microenvironment, Cervical carcinoma, Endometrial carcinoma, Uterine sarcoma, Drug repurposing, Metronomic chemotherapy, Financial toxicity

## Abstract

**Background:**

Immunotherapeutic approaches have revolutionized oncological practice but are less evaluated in gynecological malignancies. PD-1/PD-L1 blockade in gynecological cancers showed objective responses in 13–17% of patients. This could be due to immunosuppressive effects exerted by gynecological tumors on the microenvironment and an altered tumor vasculature.

In other malignancies, combining checkpoint blockade with radiation delivers benefit that is believed to be due to the abscopal effect. Addition of immune modulation agents has also shown to enhance immune checkpoint blockade efficacy. Therefore we designed a regimen consisting of PD-1 blockade combined with radiation, and different immune/environmental-targeting compounds: repurposed drugs, metronomic chemotherapy and a food supplement.

We hypothesize that these will synergistically modulate the tumor microenvironment and induce and sustain an anti-tumor immune response, resulting in tumor regression.

**Methods:**

PRIMMO is a multi-center, open-label, non-randomized, 3-cohort phase 2 study with safety run-in in patients with recurrent/refractory cervical carcinoma, endometrial carcinoma or uterine sarcoma.

Treatment consists of daily intake of vitamin D, lansoprazole, aspirin, cyclophosphamide and curcumin, starting 2 weeks before the first pembrolizumab dose. Pembrolizumab is administered 3-weekly for a total of 6 cycles. Radiation (3 × 8 Gy) is given on days 1, 3 and 5 of the first pembrolizumab dose.

The safety run-in consists of 6 patients. In total, 18 and 25 evaluable patients for cervical and endometrial carcinoma respectively are foreseen to enroll. No sample size is determined for uterine sarcoma due to its rarity.

The primary objective is objective response rate at week 26 according to immune-related response criteria.

Secondary objectives include safety, objective response rate at week 26 according to RECIST v1.1, best overall response, progression-free survival, overall survival and quality of life.

Exploratory, translational research aims to evaluate immune biomarkers, extracellular vesicles, cell death biomarkers and the gut microbiome.

**Discussion:**

In this study, a combination of PD-1 blockade, radiation and immune/environmental-targeting compounds is tested, aiming to tackle the tumor microenvironment and induce anti-tumor immunity. Translational research is performed to discover biomarkers related to the mode of action of the combination.

**Trial registration:**

EU Clinical Trials Register: EudraCT 2016-001569-97, registered on 19-6-2017. Clinicaltrials.gov: NCT03192059, registered on 19-6-2017.

**Electronic supplementary material:**

The online version of this article (10.1186/s12885-019-5676-3) contains supplementary material, which is available to authorized users.

## Background

Cervical cancer (CC) is the 3rd most common malignancy and the 4th most common cause of cancer-related deaths in women [1]. Early stage disease can often be cured with surgery and/or chemoradiation and has a good prognosis [2]. For women with extrapelvic disease, the 5-year survival rate is only 17%. For women with recurrent disease, prognosis is even worse with 5-year survival rates of less than 5% [3]. Persistent infection with human papilloma virus (HPV) is an essential step in the development of most cervical cancers [4]. In the KEYNOTE-158 trial, administration of Pembrolizumab in 98 pretreated, advanced cervical cancer patients resulted in an ORR of 13.3% (95% CI, 7.3–21.6%) and 16.0% (95% CI, 8.8–25.9%) in the whole and PD-L1-positive cohort (*n* = 81) respectively [5].

Endometrial cancer (EC) is the 5th most common malignancy in women [6]. Most ECs are diagnosed at an early stage (75%) and only a minority of these (2–15%) experience disease recurrence. When EC is diagnosed at late stages (25%) or has an aggressive histology, the chance of recurrence is very high (50%) [7]. The prognosis for patients with recurrent disease is dismal, emphasizing the high unmet need for this patient population [8]. In the phase 1b KEYNOTE-028 cohort of patients with PD-L1 positive advanced EC, 13% of patients achieved a partial response and another 13% achieved stable disease upon Pembrolizumab treatment. However, polymerase ε (POLE)-mutated and microsatellite instable (MSI) EC subgroups recently demonstrated enhanced infiltration of CD8^+^, PD-1^+^ and PD-L1^+^ immune cells [9–11]. Encouraging case reports with immune checkpoint blockade (ICB) provided proof of principle in both tumor subgroups [12, 13] and Pembrolizumab was FDA approved for all MSI+ tumors. However, POLE-mutated and MSI EC constitute only a minority of patients with recurrent EC.

Uterine sarcomas (US) are a very rare and aggressive cancer type, comprising around 3–4% of all uterine cancers. Standard treatment consists of surgery. The available cytotoxic therapies show very little clinical benefit, which is reflected by the 5-year survival rates, ranging from 57 to 65% for stage I disease to 9–26% for stage IV disease [14, 15]. PD-1 blockade in uterine sarcoma has been pursued in limited numbers of patients, but without major responses [16–18].

Clearly, immunotherapy data in CC and EC are limited. Response rates of around 15% are encouraging but not enough in this poor prognostic population. Evidence is pointing towards a crucial role of the tumor microenvironment (TME) in modulating an anti-tumor immune response, urging for combinatorial approaches to improve responses to ICB [19, 20].

Recent pre-clinical and clinical data indicate that the combination of radiotherapy (RT) with ICB showed acceptable toxicity [21, 22] and could potentiate the in situ vaccine effect of radiotherapy, mainly when given concomitant, but not sequential [23–25]. In addition, it has been described that RT induces immune cell recruitment into the tumor by releasing chemokines, thereby altering the TME [25–27].

**Fig. 1:**
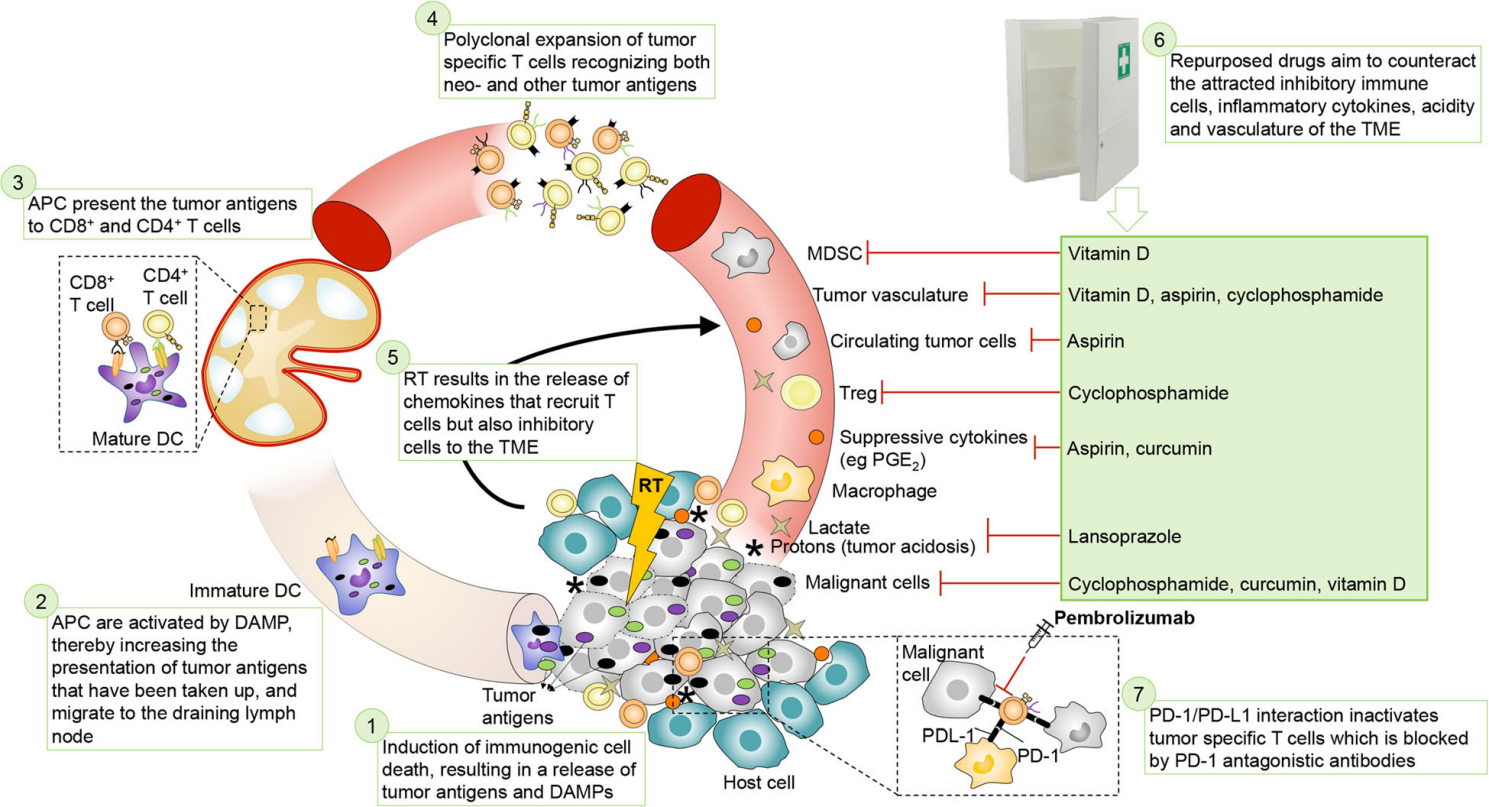
Proposed mode of action of the combination treatment. (1) Immunogenic cell death, resulting in tumor antigen (TAA) release and production of danger-associated molecular patterns (DAMPs), is induced by radiation, low-dose cyclophosphamide, curcumin and vitamin D. (2) The released TAA can be taken up by immature dendritic cells (DC) for processing through the antigen processing machinery. In the presence of DAMPs, DCs will mature and (3) migrate to tumor-draining lymph nodes to prime antigen-specific CD4^+^ and CD8^+^ T lymphocytes. (4) The primed, tumor-specific CD4^+^ and CD8^+^ T cells enter the bloodstream and undergo clonal expansion. (5) Radiation induces chemokine secretion to recruit the tumor-specific CD4^+^ and CD8^+^ T cells into the tumor to kill tumor cells. However, these chemokines also attract inhibitory cells into the TME. (6) Primed, tumor-specific CD4^+^ and CD8^+^ T cells can be inhibited by several mechanisms, which we aim to counter with repurposed drugs. Tumors maintain an acidic microenvironment by the production of protons and lactate and T cells become dysfunctional in this acidic environment. Lansoprazole will induce buffering of the extracellular pH by inhibiting vacuolar H^+^-ATPase resulting in proper functioning of T cells. The tumor vasculature is abnormal, leading to hypoxic regions in which T cells become dysfunctional. Low-dose cyclophosphamide, vitamin D and aspirin will remodel the tumor vasculature to improve T cell functioning. Tumor cells release immunosuppressive cytokines, which might be exacerbated by radiation, and leads to recruitment of myeloid-derived suppressor cells (MDSC) and tumor-associated macrophages (TAM), which inhibit T cells. Aspirin and curcumin will counter some of these cytokines such as PGE_2_ and vitamin D inhibits myeloid suppressive cells and IL-6, thereby improving T cell function. Tumors are often infiltrated by regulatory T cells (Treg) capable of inhibiting CD8^+^ T cells, which can be exacerbated by radiation. Low-dose cyclophosphamide can inhibit the suppressive activity of Treg, thereby improving the T cell response. (7) Upon activation, primed CD4^+^ and CD8^+^ T cells start to express PD-1, which can be ligated by its ligand PD-L1 on tumor cells or tumor-infiltrating immune cells, thereby limiting their effector functions. This phenomenon can be countered by pembrolizumab. Also curcumin could inhibit inflammation-mediated PD-L1. RT: radiotherapy, DAMP: danger-associated molecular pattern, APC: antigen-presenting cell, TME: tumor microenvironment, Treg: regulatory T cell, MDSC: myeloid-derived suppressor cell

In an attempt to further modulate the TME in an inexpensive manner, we added repurposed compounds (i.e. drugs approved for another indication) with (immune) modulating properties. The proposed mode of action of the combination is depicted in Fig. 1. Vitamin D is able to increase immune cell infiltration and reduce suppressive CD34^+^ cells in human tumors [28–32] and inhibits cancer stem cells [33, 34]. Aspirin acts by counter-acting COX activity [35–37] and by favoring an overall anti-angiogenic balance [38]. Lansoprazole, a proton-pump inhibitor, is added to inhibit tumor acidosis, thereby improving intratumoral immune cell function [39–42]. Low-dose cyclophosphamide exerts immunostimulatory and antiangiogenic effects [43–45]. Curcumin is a food supplement with radiosensitizing and anti-inflammatory properties [46–48].

The current phase II trial aims at exploring the therapeutic efficacy of the combination of PD-1 blockade with RT and immune/TME modulation. Considering the unknown toxicity profile of this combination, a safety run-in is performed. Given the high economical cost of PD-1 inhibitors, it is of utmost importance to identify patients who are likely to respond to these treatments beforehand. Therefore, the study is accompanied by a translational research package to evaluate immune response biomarkers in blood and tumor, characterize extracellular vesicles, explore cell death biomarkers in blood and analyze the relationship with the gut microbiome.

## Methods/design

### Objectives

#### Primary objective

To evaluate the efficacy of the treatment, which will be assessed as the objective response rate (ORR) at week 26 according to immune-related response criteria (irRC).

#### Secondary objectives

Safety according to Common Terminology Criteria for Adverse Events (CTCAE4.0), the ORR at week 26 according to RECIST criteria, the best overall response (BOR), progression-free survival (PFS), overall survival (OS) and quality of life (QoL).

#### Exploratory objectives

Exploratory objectives will be evaluated in the translational study and include evaluation of immune response biomarkers in blood and tumor biopsies, characterization of extracellular vesicles in the blood, analysis of cell death biomarkers in blood and investigation of the microbiome in feces samples (see Additional file 1).

### Trial design

The study is a Phase II multi-center, open-label, non-randomized, 3-cohort study with a safety run-in in patients with advanced and/or refractory CC, EC or US.

Even though each treatment separately has well known and tolerable safety profiles, safety of this particular combination will be determined. The safe dose is defined by 0 or 1 unmanageable dose limiting toxicity (DLT) observed in the first 6 patients within 30 days after the last RT dose irrespective of the tumor type. If 2 or more unmanageable DLTs occur, a new cohort of 6 patients will be recruited. The drug for which the adverse event is expected in the highest percentage of patients will first be adjusted in dose. If toxicity cannot be attributed to one drug, multiple drugs may become reduced in dose. Dose adjustments will continue until a safe dose is determined.

The study has an independent data safety monitoring board (DSMB) consisting of two radiotherapists, one medical oncologist, one gynecologist and one statistician. Aside from monitoring the safety, validity and integrity of the data from the study, the DSMB will evaluate the pace of recruitment and will make recommendations to the sponsor regarding the continuation, modification or termination of any or all arms of the study. Meetings will be planned every 10th patient. Bimetra Clinics, the clinical research center of the sponsor institute, will monitor this trial at several time points and at least before planned data analysis.

### Study population

#### Inclusion criteria

- Histologically confirmed CC, EC or US, refractory or persistent to chemotherapy or recurrent disease after at least 1 line of chemotherapy.
- Written informed consent.
- Age 18 years or older.
- Presence of an index lesion amenable to hypofractionated RT.
- At least 1 lesion outside the RT field for clinical response assessment.
- Willing to provide tissue from a newly obtained biopsy of a tumor lesion before and after RT if technically feasible.
- ECOG Performance status 0–2.
- Patients treated with a proton pump inhibitor or anti-coagulant should switch to the study regimen during the trial.
- Demonstrate adequate organ function as defined in Table 1.
- Female subjects of childbearing potential should have a negative pregnancy test and must use contraception.

**Table 1:** Adequate organ function laboratory values

System	Laboratory Value
Hematological	
Absolute leukocyte count	≥2500 /mcL
Absolute lymphocyte count (ALC)	≥500 /mcL
Absolute neutrophil count (ANC)	≥1500 /mcL
Platelets	≥100,000 / mcL
Hemoglobin	≥9 g/dL or ≥ 5.6 mmol/L without transfusion or EPO dependency (within 7 days of assessment)
Renal	
Serum creatinine	≤1.5 X upper limit of normal (ULN)
OR	OR
Measured or calculated^a^ creatinine clearance (GFR can also be used in place of creatinine or CrCl)	≥60 mL/min for subject with creatinine levels > 1.5 X institutional ULN
Hepatic	
Serum total bilirubin	≤ 1.5 X ULN
OR	OR
Direct bilirubin	≤ ULN for subjects with total bilirubin levels > 1.5 ULN
AST (SGOT) and ALT (SGPT)	≤ 2.5 X ULN OR ≤ 5 X ULN for subjects with liver metastases
Albumin	> 2.5 mg/dL
Coagulation	
International Normalized Ratio (INR) or Prothrombin Time (PT)	≤1.5 X ULN unless subject is receiving anticoagulant therapyas long as PT or PTT is within therapeutic range of intended use of anticoagulants
Activated Partial Thromboplastin Time (aPTT)	≤1.5 X ULN unless subject is receiving anticoagulant therapy as long as PT or PTT is within therapeutic range of intended use of anticoagulants

^a^Creatinine clearance should be calculated per institutional standard

#### Exclusion criteria

- Currently participating or has participated in a study of an investigational agent within 4 weeks of the first dose of treatment.
- Diagnosis of immunodeficiency or receiving immunosuppressive therapy
- Prior chemotherapy, targeted small molecule/antibody therapy, hormonal therapy or radiation therapy within 4 weeks prior to study.
- Known additional malignancy that requires active treatment.
- Known active central nervous system (CNS) metastases and/or carcinomatous meningitis.
- Active autoimmune disease.
- History or evidence of active, non-infectious pneumonitis.
- Active infection requiring systemic therapy.
- Prior therapy with an anti-PD-1, anti-PD-L1, or anti-PD-L2 agent.
- Known history of TB (Bacillus Tuberculosis), Human Immunodeficiency Virus (HIV), Human T cell Lymphotropic Virus (HTLV), syphilis, Hepatitis B or Hepatitis C.
- Subjects have received a live vaccine within 30 days of planned start of study therapy.

### Treatments and interventions

#### Treatments

**Fig. 2:**
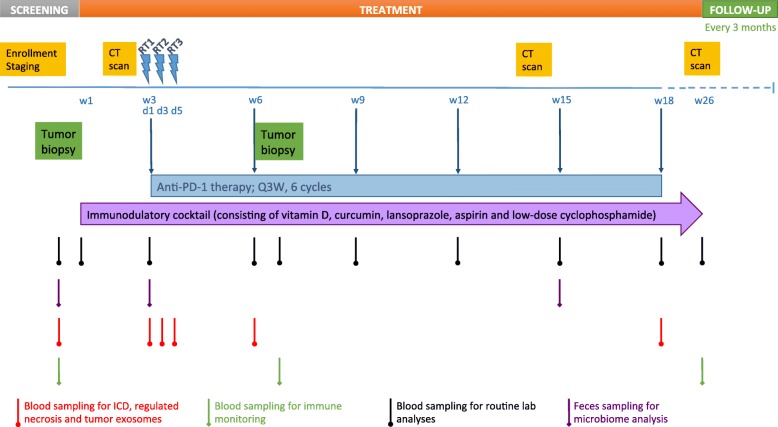
Schematic study design. RT: radiotherapy

The treatment consists of an induction phase where patients receive the combination of immune-modulatory repurposed drugs (vitamin D, aspirin, lansoprazole), metronomic chemotherapy (low-dose cyclophosphamide) and the food supplement (curcumin) for 2 weeks. Thereafter, the first dose of pembrolizumab (200 mg) is administered before the first hypofractionated RT (8 Gy) to 1 index lesion. RT is given twice (8 Gy) more with a 48-h interval. RT details are described in the Additional file 1. Pembrolizumab (200 mg) is repeated every 3 weeks for a total of 6 cycles. The immune-modulatory combination is given continuously until week 26, the time of primary endpoint measurement. A detailed overview can be found in the study scheme (Fig. 2). Patients with clinical benefit (SD, PR, CR as BOR) can continue pembrolizumab (200 mg, Q3W) for up to 2 years, according to article 34 of the Declaration of Helsinki on post-trial access to study medication. Treatment with the immunomodulatory cocktail may be continued upon investigators’ choice. To aid patient adherence to this complex treatment, patients receive a diary and only sufficient medication is provided until the next hospital visit. Concerning the immune-modulatory combination, we opted to add an extra drug or food supplement every 24 h until the complete combination is taken during the first week of the induction phase for safety reasons. Thereafter, the combination is taken as described in the treatment schedule (Table 2).

**Table 2:** Trial treatment schedule

Drug	Dose/Potency	Frequency	Time of oral intake				Route of Administration	Treatment Period	Remarks
			8 h	12 h	18 h	20 h			
Pembrolizumab (Keytruda)	200 mg	Q3W					IV infusion	Day 1 of each 3-week cycle	May be discontinued upon unacceptable toxicity Administration should be withheld for drug-related non-hematologic toxicity ≥ grade 2 (excluding fatigue) Use corticosteroids for irAE
Radiation	8 Gy	3 fractions 48 h apart						Day 1, 3 and 5 of Pembrolizumab cycle 1	See Additional file 1
Vitamin D3 (D-Cure)	2000 IU (50 μg) ^a^	Daily			X		Oral	Daily from day −14	Intake of other vitamin D preparations should be stopped Blood should be monitored for calcium and phosphate Caution in patients receiving digitalis preparations
Lansoprazole (Lansoprazole Teva)	180 mg (uneven weeks); 30 mg (even weeks) ^b^	Daily		X		X ^c^	Oral	Daily from day −12	Needs to be taken at least 30 min before a meal Patients taking any kind of proton pump inhibitors should switch to Lansoprazole Teva throughout the study period Must be taken 2 h apart from all other orally administered drugs
Aspirin (Sedergine)	325 mg ^d^	Daily	X				Oral	Daily from day −11	Patients receiving any other anti-coagulant therapy should switch to Sedergine throughout the study
Cyclophosphamide (Endoxan)	50 mg ^e^	Daily	X				Oral	Daily from day −10	Monitor leukocyte levels regularly during treatment Do not administer to patients with leukocyte levels < 2500/μl and/or thrombocyte levels of < 50,000/μl Consumption of grapefruit and its derivatives is counter-advised
Curcumin (CurcuPhyt) (Food supplement, NIMP)	2 g ^f^	Daily	X		X		Oral	Daily from day −13	Patients are suggested to not take H2 Beta blockers (beta-adrenoreceptor antagonists) Proton pump inhibitors should be consumed 2 h apart Patients should not consume other turmeric containing food

^a^30 drops should be resolved in fat-containing drink, e.g. milk

^b^ Capsules should be swallowed entirely with fluid

^c^ uneven weeks only

^d^ Effervescent tablets should be dissolved in water

^e^ Tablets should be taken with a large amount of fluid.

^f^ Capsules should be taken with a meal and not be chewed on

#### Interventions

The study flow chart (Fig. 2 and Additional file 1: Table S1) tabulates the timing of all scheduled drug administrations, blood samplings and tumor imaging procedures throughout the study to document safety and efficacy of the treatment.

For the exploratory translational research, blood, biopsy and feces collections take place at different time points during the study.

The details of the exploratory translational research analyses are described in detail in the Additional file 1.

### Sample size

The total sample size consists of both evaluable and non-evaluable patients. Evaluable patients are defined as patients who received at least all components of the treatment, being 2 weeks of immunomodulatory combination, 1 dose of pembrolizumab and the 3 radiotherapy fractions. The study will continue until the necessary number of evaluable patients is reached. Patients included in the safety run-in will be part of the efficacy analysis to fasten inclusion. However, each cohort will be analyzed separately and no comparison between disease types will be made. The sample size is calculated based on a two-stage design using exact binomial tests. Estimated numbers of CC and EC patients needed to achieve the primary study objective and the clinical and statistical assumptions made can be found in Table 3. For the US group, no sample size calculation is performed due to the rarity of the disease. However, to provide these patients with a possibly effective treatment option, these patients will be allowed on study. These data will provide an indication for possible further development of this treatment in uterine sarcoma.

**Table 3:** Statistical sample size calculation

	Null hypothesis (H_0_)	Total number of patients required	Number of responses needed for H0 rejection	Alternative hypothesis	Type I error	power
Cervical cancer	π > 10%	18	5	π =35%	5%	80%
Endometrial carcinoma	π > 10%	25	6	π =30%	5%	79.3%
Uterine sarcoma	No sample size calculation is performed due to the rarity of the disease, but these patients will be allowed on the study					

### Data analysis

The CC and EC groups will be analyzed separately. No correction for multiple testing will be applied. Only descriptive statistics will be provided for the uterine sarcoma group.

#### Primary endpoint

Patients with complete or partial response at week 26 according irRC criteria will be regarded as responders. Patients for whom the scheduled tumor imaging at week 26 is not available will be considered to be a non-responder for the primary analysis.

The null hypothesis that the true response rate π is 10% will be tested against a one-sided alternative (Ha: π > 10%). A point estimate with a 90% confidence interval will be reported.

#### Secondary endpoints

##### Safety

The number of unmanageable dose limiting toxicities will be reported for the run-in period and the main trial. The number of patients with AEs, serious AEs (SAEs) and treatment-related AEs will be summarized by system organ class and preferred term and by worst toxicity grade. Laboratory safety and other safety assessments will be described descriptively by visit and the difference with the baseline visit for all other visits for each group separately.

##### Response rate at week 26 according to RECIST

The ORR at week 26 according to RECIST will be analyzed similarly as the primary endpoint.

##### Best overall response

The BOR is defined as the best response (confirmed complete or partial response, per RECIST v1.1) recorded from the start of the study treatment at any time during the study taking into account any requirement for confirmation. It will be analyzed similarly as the primary endpoint.

##### Progression-free survival

The PFS, defined as the time from start of treatment until progression or last follow-up, will be analyzed as interval censored data by means of the Turnbull estimate. Patients without progression will be censored at their last visit. At weeks 26, 52, 75, 104, 130 and 156 the proportion of progression-free patients will be estimated with a 95% confidence interval and median PFS will be calculated.

##### Overall survival

The OS, defined as the time from start of treatment until death, will be analyzed by means of a Kaplan-Meier estimate. Patients who survived will be censored at their last visit. At weeks 26, 52, 75, 104, 130 and 156 the proportion of patients surviving will be estimated with a 95% confidence interval and median OS will be calculated.

##### Quality of life

Quality of life will be measured by FACT-Cx questionnaire for the CC group and by the FACT-G questionnaire for the EC and US groups. Descriptive statistics of the total score at each visit and the difference with the baseline visit for all other visits will be reported.

### Exploratory endpoints

The explorative endpoints will be described descriptively by visit and the difference with the baseline visit for all other visits for each group separately. They will be related to the primary endpoint, ORR at week 26 according to RECIST, BOR, PFS and OS.

### Subgroup analyses

For all primary and secondary endpoints subgroup analysis is planned. This is exploratory and not statistically powered. For CC, squamous versus non-squamous histology, HPV-positive versus HPV-negative tumors and PD-L1 status will be evaluated. For EC, endometroid versus non-endometroid histology, grade 1 versus grade 2/3 tumors, MSI versus MSS, hormone receptor positive versus negative, PTEN deficient versus wild-type, and wild-type POLE versus proofreading mutant POLE status will be analyzed. For both CC and EC, correlation with absolute lymphocyte count before treatment and at 0, 1 and 3 months after the first pembrolizumab administration will be evaluated. Subgroup analysis is based on the etiology of each cancer type.

Comparison for all primary and secondary endpoints between the subgroups will be done by a Fisher’s Exact test.

Comparison for PFS between the subgroups will be done by means of the generalized logrank test of Sun.

Comparison for OS between the subgroups will be done by means of the logrank test.

## Discussion

Although ICB has led to remarkable response rates in some subtypes of uterine cancers, the majority of patients with recurrent CC, EC or US do not benefit from single-agent ICB, urging for the development of more effective therapeutic regimens for these patients. We hypothesize that combining PD-1 blockade with radiotherapy and additional immune modulators might result in clinical responses in about one third of patients. The combination with repurposed compounds is also of important economical value, as opposed to combinations with novel, expensive drugs that are posing financial toxicity to the healthcare systems [49–53]. In the current study, we aim to assess the efficacy of this novel combination treatment in recurrent CC, EC and US. The innovation of this study originates from the combined use of 7 treatments to simultaneously act on tumor metabolism, angiogenesis and anti-tumor immunity. In addition, the translational research focused on immunologic markers, extracellular vesicles, cell death biomarkers and alterations in the gut microbiome might be suitable to identify mechanisms of response and resistance to therapy, resulting in predictive biomarkers for efficacy and improved patient selection in future clinical applications.

## Additional file

Additional file 1: 1. Additional data. 2. Additional Tables. **Table S1:** Trial flowchart. **Table S2:** Planned immunological analyses. (DOCX 95 kb)

## Abbreviations

ACTH: Adrenocorticotropic hormone
AE: Adverse event
ALC: Absolute lymphocyte count
ALT: Alanine aminotransferase
ANA: Anti-nuclear antibodies
ANC: Absolute neutrophil count
APC: Antigen-presenting cell
aPTT: Activated partial thromboplastin time
AST: Aspartate aminotransferase
BOR: Best overall response
CBC: Complete blood count
CNS: Central nervous system
COX: Cyclooxygenase
CR: Complete response
CRP: C-reactive protein
CT: Computer tomography
CTCAE: Common Terminology Criteria for Adverse Events
CY: Cyclophosphamide
DAMPs: Danger-associated molecular patterns
DC: Dendritic cell
DLT: Dose limiting toxicity
DSMB: Data safety monitoring board
EC: Endometrial cancer
ECG: Electrocardiogram
ECOG: Eastern cooperative oncology group
EPO: Erythropoietin
FDA: Food and drug administration
GFR: Glomerular filtration rate
Gy: Gray
HCG: Human choriongonadotrophin
HCV: Hepatitis C virus
HIV: Human immunodeficiency virus
HPV: Human papilloma virus
HTLV: Human T cell Lymphotropic Virus
ICB: Immune checkpoint blockade
IL: Interleukin
INR: International normalized ratio
irAE: Immune-related adverse event
irRC: Immune-related response criteria
LDH: Lactate dehydrogenase
LH: Luteinizing hormone
mAb: Monoclonal antibody
MDSC: Myeloid-derived suppressor cells
MSI: Microsatellite instable
MSS: Microsatellite stable
NIMP: Non-investigational medicinal product
ORR: Overall response rate
OS: Overall survival
PD: Programmed death
PD-L: Programmed death ligand
PFS: Progression-free survival
PGE: Prostaglandin E
POLE: Exonuclease domain of polymerase ε
PR: Partial response
PT: Prothrombin time
PTEN: Phosphatase and tensin homolog
Q3W: Every 3 weeks
QoL: Quality of life
RECIST: Response Evaluation Criteria in Solid Tumors
RT: Radiotherapy
SAE: Serious adverse event
SD: Stable disease
SGOT: Serum glutamic oxaloacetic transaminase
SGPT: Serum glutamic pyruvic transaminase
TAA: Tumor-associated antigen
TAM: Tumor-associated macrophages
TB: Tuberculosis bacillus
TIL: Tumor-infiltrating lymphocyte
TME: Tumor microenvironment
TPO: Thyreoperoxidase
Treg: Regulatory T cells
TSH: Thyroid stimulating hormone
TSI: Thyroid-stimulating immunoglobulin
ULN: Upper limit of normal
