# Supplementary material for: PRIMMO study protocol: a phase II study combining PD-1 blockade, radiation and immunomodulation to tackle cervical and uterine cancer
Source: BMC Cancer. 2019 May 28;19:506. doi: 10.1186/s12885-019-5676-3 (PMC6537207; doi:10.1186/s12885-019-5676-3)
Supplement: Supplementary file 1 — 1. Additional data. 2. Additional Tables. Table S1: Trial flowchart. Table S2: Planned immunological analyses. (DOCX 95 kb) [file 12885_2019_5676_MOESM1_ESM.docx]

***Additional File 1***

**PRIMMO study protocol: a phase II study combining PD-1 blockade, radiation and immunomodulation to tackle cervical and uterine cancer**

Sandra Tuyaerts^1*^, An M. T. Van Nuffel^2^, Eline Naert^3^, Peter Van Dam^4^, Peter Vuylsteke^5^, Alex De Caluwé^6^, Piet Dirix^7^, Lien Lippens^3^, Emiel De Jaeghere^3^, Frédéric Amant^1,8^, Katrien Vandecasteele^9^, Hannelore Denys^3*^

Correspondence:

sandra.tuyaerts@kuleuven.be

hannelore.denys@uzgent.be

# Additional Data

## Detailed radiotherapy description

During the first cycle of pembrolizumab treatment, stereotactic body radiation therapy (SBRT) will be delivered to one index lesion. A total dose of 24 Gy will be delivered in three fractions of 8 Gy, with each fraction 48h apart. A lesion causing symptoms or discomfort to the patient is preferred to be chosen as index lesion, a bony lesion is to be avoided if other lesions are amenable. All patients will be immobilized in a comfortable and appropriate position to irradiate the index lesion. Support and/or immobilization devices can be used to increase patient comfort or to ensure set-up reproducibility. The planning CT scan should be acquired with the patient in the same position and using the same immobilization/support device(s) as for treatment. Planning CT scan (with IV contrast, unless contraindicated) will be done at ≤ 3 mm intervals encompassing the region of interest with sufficient margin for treatment planning, a typical scan length should extend at least 10 cm superior and inferior beyond the treatment field borders. Lung and liver tumor sites will be simulated with 4D-CT or a similar established technique, taking into account breathing-motion. For abdominal or pelvic tumor sites bowel contrast should be used for planning CT scan. The Gross Tumor Volume (GTV) is defined as all known gross disease encompassing the selected index lesion. The GTV will consist of the index lesion as visualized on CT. No Clinical Target Volume (CTV) will be delineated. The Internal Tumor Volume (ITV) is defined for mobile index lesions (lung or liver) at the discretion of the treating physician. A 4-D CT scan should be acquired in order to account for the motion of the lesion during respiration. The ITV will be defined as the union of the visualized index lesion on all gated CT data sets. The Planning Target Volume (PTV) will be created by using a 3-dimensional margin on the GTV or ITV (if available) to allow for daily set-up variance and organ motion. Margins depend on the site irradiated with typically 2 mm margins for bony lesions and 5 mm for other sites. Organs at risk (OAR) are contoured as visualized on the planning CT with IV-contrast. The OAR depend on the localization of the metastasis and should at least include all OAR (lying within the scanned range on the planning CT scan) for which dose constraints are described in the report of the AAPM task group 101[1]. A Planning Organ at Risk Volume (PRV) expansion of typically 2 mm will be added to the OAR for setup uncertainty or organ motion. For mediastinum, liver, heart and kidney a PRV margin of 5mm should be used. All dose constraints apply to this PRV. It is strongly recommended that dose constraints be not exceeded. If a dose constraint cannot be achieved due to overlap of the target with an OAR, the target coverage can be compromised in order to meet the constraint. Treatment will be prescribed to the periphery of the target, i.e. 80% of the dose (=24 Gy), should cover 90% of the PTV. The OAR dose constraints will be in accordance with the recommendations from the report of the AAPM task group 101. In case of violation of dose constraints to the surrounding OARs, the prescription can be adapted accordingly. A dose inhomogeneity in the PTV overlapping with a PRV is allowed but 90% of the GTV should receive at least 24 Gy. Maximum PTV dose up to 160% is allowed but all dose > 105% (31.5 Gy) should lie within the PTV. Dose falloff outside the PTV extending into normal tissue structures must be rapid in all directions and one should target a dose fall-off of 50% off the prescribed dose (12 Gy) within 3 cm outside the PTV.

## Biomarker study ancillary to the PRIMMO trial

*Objectives*

The main objectives of this translational research study are to (1) unravel the contribution of each treatment component to therapeutic efficacy and (2) identify predictive biomarkers for this novel therapeutic combination.

To this end, we plan to investigate:

- Immune response biomarkers
- Extracellular vesicles
- Cell death biomarkers
- Gut microbiome composition

*Immune response biomarkers*

Due to the revival of cancer immunotherapy, monitoring the immune response as well as tumor responses following these therapies is of utmost important to the advancement of this field, and the identification of predictive biomarkers as well as early markers of response to new treatments are important goals of ongoing research in order to broaden the impact of these therapeutics [2]. At present no definitive biomarkers have been validated that can be used to predict which patients are most likely to have a clinical benefit. A major complicating factor is the site where immune monitoring should be performed: peripheral blood is readily accessible but the relationship between the peripheral immune system and immune responses in the TME is not well understood [3]. Moreover, even between primary tumors and metastatic sites, the employed immunological mechanisms have been suggested to be distinct [4, 5]. Next, the immune response is highly dynamic in nature, which poses a significant challenge to immunomonitoring approaches. Therefore, timing of the collection of biological samples for immune response monitoring is of high importance. According to the PRIMMO protocol, blood for immune response biomarkers is collected at screening, during treatment cycle 2 and after treatment cycle 6 (end of treatment). Thereafter, for patients that continue on treatment, yearly follow-up samples will be collected or at time of progression. In patients where it is technically feasible, tumor biopsies will be collected at screening and during treatment cycle 2, see also figure 2 (main manuscript) and additional table S1. Sampling at baseline would allow the identification of predictive or prognostic biomarkers for patient stratification. The sampling during cycle 2 is based on (1) the hypothesis that at this time point T cells would have infiltrated the irradiated lesion and (2) previously published findings that immune profiling in early on-treatment biopsies is highly predictive of response to PD-1 blockade in melanoma [6]. The blood sampling at end of treatment serves to assess the effect of treatment on the induced immune response and to correlate findings with clinical outcome.

From blood, serum, plasma, RNA, DNA and peripheral blood mononuclear cells will be isolated and a broad set of immunological analytes (summarized in additional table S2, with their rationale) will be measured together with immunophenotyping of immune cells and T cell functional assays.

The fresh tumor biopsies, depending on the available amount of tumor material, will be divided for embedding in paraffin, RNA/DNA isolation, TIL culture and single-cell suspension preparation for flow cytometric analyses. On these samples, we will perform longitudinal analysis of the immune infiltrate [7], PD-L1 expression [8], TCR repertoire [8], mutational load [9] and T cell functional assays. An archival tumor biopsy from all patients will be used to assess immune cell infiltration, PD-L1 status, PTEN status, ER/PR expression, MSI status and POLE status because it has been shown previously that these could potentially correlate with efficiency of PD-1 blockade.

These data will allow us to identify whether the treatment has an impact of the intratumoral immune response diversity and whether these changes are reflected in the blood. Moreover, by correlating immunological changes with known clinical findings, we hope to find predictive biomarkers for patient selection in future trials.

*Extracellular vesicles (EVs)*

Cells communicate with each other by secreting a range of different components. On the one hand soluble factors (e.g. hormones, growth factors) but also complexes composed of proteins, lipids and/or nucleic acids (e.g. protein complexes, lipoproteins, extracellular vesicles (EVs)). EVs are nanovesicles, secreted in high number by cancer cells, with a diameter of 50-1000 nm and can be subdivided in microvesicles and exosomes on the basis of their cellular origin [10]. They are composed of a double layered lipid membrane, some specific lipids, membrane as well as intraluminal proteins and nucleic acids [11, 12]. In this way they can be seen as packages filled with information that can be transported from donor cell to recipient cell. Donor and recipient cells can be located close to each other, e.g. in the same organ, but EVs can also end up in the blood stream and can be transferred to distant organs where they can support the development of a pre-metastatic niche [13]. As EVs are secreted by cancer cells in high numbers and their composition reflects the parent cell, characterization of EVs may provide valuable information about the tumor. EVs entering the bloodstream have shown promising predictive or prognostic value for patient follow-up in different cancer types [14-17]. As EVs contain proteins, lipids and nucleic acids, each of these classes of components can lead to a completely different way of using EVs as a biomarker [18]. EVs also have the advantage that they can be obtained in a noninvasive way, by liquid biopsy sampling, as EVs can be found in blood, urine, breast milk, saliva, sperm, amniotic fluid etc. [19].

According to the PRIMMO protocol, blood for extracellular vesicle analysis is collected at six time points: at screening, three times during treatment cycle 1: within one hour after the first radiotherapy treatment, within one hour before the second and before the third radiotherapy treatment, at day 1 of treatment cycle 2 and at day 1 of cycle 6. EVs will be isolated from platelet free plasma by our in house created protocol using size exclusion chromatography and OptiPrep density gradient to end up with pure EVs. Isolated EVs will be analyzed for numbers and alterations on the level of transcriptome and proteome. After biostatistical and bioinformatical analysis a correlation of EV profiles with corresponding known therapy specific changes will be carried out. In this way we would like to identify a biomarker that could predict the response of the patient with cervical carcinoma, endometrial carcinoma or uterine sarcoma before or early during treatment. Ultimately, leading to exclusion of patients not benefitting from the treatment.

*Cell death biomarkers*

Since some time, it has become clear that the therapeutic potential of RT does not only exist of local and direct cytotoxicity but also indirect local and long-range abscopal (systemic) effects [20, 21]. Hereby RT turns the tumor into an “in-situ vaccine” through the mechanism of ICD [22]. Induction of ICD by RT is dependent on radiation dose and fractionation. RT used at immunogenic doses leads to accumulation of cytosolic double-stranded DNA (dsDNA) in cancer cells, which activates type I IFN via the cGAS/STING pathway. Radiation doses per fraction above 10-12 Gy induce TREX1 upregulation in the irradiated tumor cells and a concominant degradation of IFN-stimulatory cytosolic dsDNA [23, 24]. Immunogenic cell death implicates the release of damage-associated molecular patterns (DAMPs) and tumor antigens through a disintegrated cell plasma membrane [25, 26], linking ICD with necrosis rather than apoptosis (a programmed cell death in which the cell membrane remains intact). Necrosis, which for long had been considered as a purely accidental cell death mode, can be programmed or induced by finely regulated signal transduction pathways, called regulated necrosis (RN). Necroptosis and ferroptosis are, amongst others, 2 forms of RN [27]. Necroptosis, recently described to be involved in radiation induced programmed cellular death can be present in apoptotic/caspase-deficient conditions and may compensate for the resistance to apoptosis (a major cause of therapy resistance) [28]. Ferroptosis is a regulated form of iron-dependent cell death genetically, biochemically, and morphologically distinct from other cell death modalities and is driven by loss of activity of the lipid repair enzyme glutathione peroxidase 4 (GPX4) and subsequent accumulation of lipid-based reactive oxygen species (ROS), particularly lipid hydroperoxides [29]. Secondary necrosis (SN) occurs in apoptotically dying cells which cannot be properly and timely engulfed by neighboring cells or professional (macro)phagocytes in case of massive apoptosis [20].

Key question from immuno-radiotherapeutic point of view is how to achieve optimal cell death required for ICD and if cell death related products could be used as biomarker for response prediction. However, first of all we need to explore which cell death modalities are involved in ICD, since this is most probably *NOT* apoptotic (together with mitotic catastrophe and senescence considered to be the principal mechanism of radiation induced cell death for years) [20, 30] but rather a regulated or secondary necrotic cell death.

According to the PRIMMO protocol, blood for cell death biomarkers is collected at the same time as EVs blood collection, being at screening, 3 times during treatment cycle 1 (within 1 hour after the first and within one hour before the 2nd and 3rd RT treatment) and at day 1 of treatment cycle 2 and 6.

We will investigate the impact of treatment on release of danger signals (HMGB-1, CRT, mtDNA) and cell death related proteins (cytochrome C, IL1, cytokeratin-18, phosphorylated mixed lineage kinase domain-like protein, activated caspase-3, drop in GPX4 expression) to distinguish between apoptosis, SN and several kinds of regulated necrosis. We hypothesize that response to treatment will be associated with presence or change of danger signals and/or cell death markers in the blood, this will allow us to predict response to treatment soon after start of the RT treatment.

*Gut microbiome*

The intestinal epithelial barrier hosts a broad range of commensal bacteria, other microorganisms and their genomes, collectively referred to as the gut microbiome (GM) [31, 32]. Factors reported to influence the composition of the GM include age [33], host genetics [34], tobacco smoking [35], systemic antibiotic therapy [36] and nutrition [37]. In most cases the GM is beneficial for the development, health and survival of the organism [38]. These microbes contribute to a broad range of physiological functions including nutrient metabolism, enteric and systemic antimicrobial protection, hematopoiesis and other functions systemically [39, 40]. A growing body of literature, however, underscores the association between the GM and cancer initiation, invasion and metastasis both at colonized and sterile tissues [40-42].

Interestingly, the relation between the GM and response to cancer therapy is being increasingly recognized [43]. Overall, the evidence from human, mouse and *in vitro* studies supports an intimate relationship between the GM and the response to chemotherapy including cyclophosphamide [44], oxaliplatin [45], 5-fluorouracil [46], gemcitabine [47, 48], irinotecan [49] and methotrexate [50, 51]. More recently, it has been shown that commensal bacteria control cancer response to immune checkpoint therapy such as PD-L1 and CTLA-4 blockade by modulating the host immune response [52-54]. In a pivotal multi-institutional study, Gopalakrishnan and colleagues [55] evaluated the composition of the GM in 43 patients treated with PD-1 blockade for metastatic melanoma. According to RECIST criteria, participants were classified as either responders or non-responders. The study team demonstrated significantly different enterotypes in the gut of responders versus non-responders. Of interest, responders harbored a significantly higher bacterial diversity (p<0.01) and had increased concentration of the *Ruminococcaceae* bacteria (p<0.01) when compared with their non-responding counterparts. Other results from patients receiving checkpoint inhibitors for metastatic renal cell carcinoma have revealed that broad-spectrum antibiotics have a significant negative effect on progression-free survival. As the authors concluded [56], additional research is warranted to determine whether the alteration of the GM is responsible for this finding. In brief, this large number of preclinical and clinical studies suggests that, however significant progress in the field of the oncomicrobiome has been made, this area of research is nascent and the research is preliminary.

According to the PRIMMO protocol, fecal samples are collected at three different time points to explore connections between the composition of the GM and therapy response. Their relevance is twofold. First, only a minority of patients typically responds to PD-1 blockade [57, 58], at the expense of high treatment costs. Therefore, patient selection based on the gut signature holds great promise as a tool to identify patients that are most likely to respond to PD-1 blockade. Second, manipulation of the GM could prove to be a future actionable strategy to enhance therapeutic responses to immune checkpoint blockade [59, 60].

# Additional tables

## Additional file 1: Table S1 Trial flowchart

| **Trial Period:** | **Screening** | **Treatment** | | | | | | | | | | | | | | | | | | | | | | | | | | **Follow-up** | | |
| --- | --- | --- | --- | --- | --- | --- | --- | --- | --- | --- | --- | --- | --- | --- | --- | --- | --- | --- | --- | --- | --- | --- | --- | --- | --- | --- | --- | --- | --- | --- |
| Scheduling Window | Week -4 - 0 | Week 1 D-14 | Week 1 D-13 | Week 1 D-12 | Week 1D-11 | Week 1 D-10 | Week 1 D-9 – D0 | Week 2 | Week 3 D1 | Week 3 D3 | Week 3 D5 | Week 3-5 | Week 6 D1 | Week 7 D8 | Week 6-8 | Week 9 D1 | Week 9-11 | Week 12 D1 | Week 12-14 | Week 14 D15 | Week 15 D1 | Week 15-17 | Week 18 D1 | Week 18-20 | Week 21 D1 | Week 21-25 | Week 26 / discontinuation | 30 days post discon | 12 weeks post discon | Every 3 months |
| **Treatments** | | | | | | | | | | | | | | | | | | | | | | | | | | | | | | |
| Pembrolizumab |  |  |  |  |  |  |  |  | X |  |  |  | X |  |  | X |  | X |  |  | X |  | X |  |  |  |  |  |  |  |
| Radiotherapy |  |  |  |  |  |  |  |  | X | X | X |  |  |  |  |  |  |  |  |  |  |  |  |  |  |  |  |  |  |  |
| D-Cure |  | X | X | X | X | X | X |  |  |  |  | X |  |  | X |  | X |  | X |  |  | X |  | X |  | X |  |  |  |  |
| Lansoprazole Teva |  |  |  | X | X | X | X |  |  |  |  | X |  |  | X |  | X |  | X |  |  | X |  | X |  | X |  |  |  |  |
| Sedergine |  |  |  |  | X | X | X |  |  |  |  | X |  |  | X |  | X |  | X |  |  | X |  | X |  | X |  |  |  |  |
| Endoxan |  |  |  |  |  | X | X |  |  |  |  | X |  |  | X |  | X |  | X |  |  | X |  | X |  | X |  |  |  |  |
| **Food supplement** | | | | | | | | | | | | | | | | | | | | | | | | | | | | | | |
| CurcuPhyt |  |  | X | X | X | X | X |  |  |  |  | X |  |  | X |  | X |  | X |  |  | X |  | X |  | X |  |  |  |  |
| **Administrative Procedures** | | | | | | | | | | | | | | | | | | | | | | | | | | | | | | |
| Informed Consent | X |  |  |  |  |  |  |  |  |  |  |  |  |  |  |  |  |  |  |  |  |  |  |  |  |  |  |  |  |  |
| Inclusion/Exclusion Criteria | X |  |  |  |  |  |  |  |  |  |  |  |  |  |  |  |  |  |  |  |  |  |  |  |  |  |  |  |  |  |
| Demographics and Medical History | X |  |  |  |  |  |  |  |  |  |  |  |  |  |  |  |  |  |  |  |  |  |  |  |  |  |  |  |  |  |
| Cancer history | X |  |  |  |  |  |  |  |  |  |  |  |  |  |  |  |  |  |  |  |  |  |  |  |  |  |  |  |  |  |
| Prior Medication | X |  |  |  |  |  |  |  |  |  |  |  |  |  |  |  |  |  |  |  |  |  |  |  |  |  |  |  |  |  |
| Concomitant Medication |  | X |  |  |  |  |  |  | X |  |  |  | X | X |  | X |  | X |  |  | X |  | X |  | X |  | X | X |  |  |
| Patients diary |  |  |  |  |  |  |  |  | X |  |  |  | X |  |  | X |  | X |  |  | X |  | X |  | X |  | X |  |  |  |
| Post-study anticancer therapy status |  |  |  |  |  |  |  |  |  |  |  |  |  |  |  |  |  |  |  |  |  |  |  |  |  |  |  | X | X | X |
| Survival Status |  |  |  |  |  |  |  |  |  |  |  |  |  |  |  |  |  |  |  |  |  |  |  |  |  |  |  | X | X | X |
| **Clinical Procedures/Assessments** | | | | | | | | | | | | | | | | | | | | | | | | | | | | | | |
| Review Adverse Events |  |  |  |  |  |  |  |  | X |  |  |  | X | X |  | X |  | X |  |  | X |  | X |  |  |  | X | X | X |  |
| Adverse event follow up |  |  |  |  |  |  |  |  |  |  |  |  |  |  |  |  |  |  |  |  |  |  |  |  |  |  |  |  |  | X |
| ECG | X |  |  |  |  |  |  |  |  |  |  |  |  |  |  |  |  |  |  |  |  |  |  |  |  |  |  | X |  |  |
| Physical Examination | X | X |  |  |  |  |  |  | X |  |  |  | X | X |  | X |  | X |  |  | X |  | X |  |  |  | X | X | X |  |
| Clinical complaints |  | X |  |  |  |  |  |  | X |  |  |  | X |  |  | X |  | X |  |  | X |  | X |  | X |  | X | X | X | X |
| Height | X |  |  |  |  |  |  |  |  |  |  |  |  |  |  |  |  |  |  |  |  |  |  |  |  |  |  |  |  |  |
| Vital Signs & Weight | X | X |  |  |  |  |  |  | X |  |  |  | X | X |  | X |  | X |  |  | X |  | X |  |  |  | X | X |  |  |
| ECOG status | X | X |  |  |  |  |  |  | X |  |  |  | X | X |  | X |  | X |  |  | X |  | X |  |  |  | X | X |  |  |
| CT scan to plan radiotherapy |  |  |  |  |  |  |  | X |  |  |  |  |  |  |  |  |  |  |  |  |  |  |  |  |  |  |  |  |  |  |
| Radiotherapy Toxicity |  |  |  |  |  |  |  |  |  |  |  |  |  |  |  |  |  | X |  |  |  |  |  |  |  |  |  |  |  |  |
| **Laboratory Procedures/Assessments: analysis performed by LOCAL laboratory** | | | | | | | | | | | | | | | | | | | | | | | | | | | | | | |
| Pregnancy Test | X |  |  |  |  |  |  |  |  |  |  |  |  |  |  |  |  |  |  |  |  |  |  |  |  |  |  |  |  |  |
| PT/INR and aPTT | X |  |  |  |  |  |  |  |  |  |  |  |  |  |  |  |  |  |  |  |  |  |  |  |  |  |  |  |  |  |
| CBC with Differential | X | X |  |  |  |  |  |  | X |  |  |  | X | X |  | X |  | X |  |  | X |  | X |  | X |  | X | X |  |  |
| Serum Chemistry | X | X |  |  |  |  |  |  | X |  |  |  | X | X |  | X |  | X |  |  | X |  | X |  | X |  | X | X |  |  |
| Urinalysis | X |  |  |  |  |  |  |  |  |  |  |  |  |  |  |  |  |  |  |  |  |  |  |  |  |  |  |  |  |  |
| Endocrine panel | X | X |  |  |  |  |  |  | X |  |  |  |  |  |  | X |  |  |  |  | X |  |  |  | X |  | X | X |  |  |
| CA125 | X | X |  |  |  |  |  |  | X |  |  |  |  |  |  | X |  |  |  |  | X |  |  |  | X |  | X | X |  |  |
| **Efficacy Measurements** | | | | | | | | | | | | | | | | | | | | | | | | | | | | | | |
| Tumor Imaging | X |  |  |  |  |  |  |  |  |  |  |  |  |  |  |  |  |  |  | X |  |  |  | X |  |  | X | X | X | X |
| QoL questionnaire | X |  |  |  |  |  |  |  |  |  |  |  |  |  |  |  |  | X |  |  |  |  |  |  |  |  | X |  | X |  |
| **Tumor Biopsies/Archival Tissue Collection/Correlative Studies Blood** | | | | | | | | | | | | | | | | | | | | | | | | | | | | | | |
| Archival or Newly Obtained biopsy | X |  |  |  |  |  |  |  |  |  |  |  |  | X |  |  |  |  |  |  |  |  |  |  |  |  |  |  |  |  |
| Immune Monitoring Blood | X |  |  |  |  |  |  |  |  |  |  |  |  | X |  |  |  |  |  |  |  |  |  |  |  |  | X |  |  |  |
| Biomarkers Blood | X |  |  |  |  |  |  |  | X | X | X |  | X |  |  |  |  |  |  |  |  |  | X |  |  |  |  |  |  |  |
| Feces sample | X |  |  |  |  |  |  |  | X |  |  |  |  |  |  |  |  |  |  |  | X |  |  |  |  |  |  |  |  |  |

## Additional file 1: Table S2 Planned immunological analyses

| **Analyte** | **Rationale** | **Site of assessment** |
| --- | --- | --- |
| TCR repertoire analysis | T cell response | PBMCs and tumor |
| Tumor-specific T cells (intracellular cytokines, perforin, granzyme, CD107) | T cell function | PBMCs |
| T cell phenotype | T cell memory/activation status | PBMCs and tumor |
| Cytokine/chemokine assessment | Humoral response  Systemic cytokine response | Blood – serum or plasma |
| Kynurenine, Tryptophan | IDO1 status | Blood – serum or plasma |
| IDO1 expression | IDO1 status | Tumor |
| CD3, CD8, CD45RO, CD68, CD163, FoxP3, granzyme B, NKp46 | TIL infiltration | Tumor |
| sPD-L1 | Potential checkpoint blockade biomarker | Blood – serum or plasma |
| sMICA | Radiation-induced T and NK activation | Blood – serum |
| MDSC | MDSC frequency | Blood and tumor |
| Arginase-1, PGE_2_, TGFβ | MDSC function | Blood – serum or plasma |
| COX-2 | COX-2-driven inflammation | Tumor |
| DC phenotype | DC subsets | PBMCs and tumor |
| NK cell phenotype | NK cell subsets | PBMCs and tumor |
| RNA sequencing | Tumor mutational burden  Differential gene expression | PBMCs and tumor |
| MSS/MSI | MMR status | Tumor |
| PTEN | PTEN status | Tumor |
| PD-L1 | PD-L1 status | Tumor |
| POLE | POLE status | Tumor |
| HPV | HPV infection | Tumor |
| Hormone receptors (ER, PR) | Hormone receptor expression | Tumor |

# References

1. Benedict SH, Yenice KM, Followill D, Galvin JM, Hinson W, Kavanagh B, Keall P, Lovelock M, Meeks S, Papiez L, et al: **Stereotactic body radiation therapy: the report of AAPM Task Group 101.** *Med Phys* 2010, **37:**4078-4101.

2. Kohrt HE, Tumeh PC, Benson D, Bhardwaj N, Brody J, Formenti S, Fox BA, Galon J, June CH, Kalos M, et al: **Immunodynamics: a cancer immunotherapy trials network review of immune monitoring in immuno-oncology clinical trials.** *J Immunother Cancer* 2016, **4:**15.

3. Stroncek DF, Butterfield LH, Cannarile MA, Dhodapkar MV, Greten TF, Grivel JC, Kaufman DR, Kong HH, Korangy F, Lee PP, et al: **Systematic evaluation of immune regulation and modulation.** *J Immunother Cancer* 2017, **5:**21.

4. Riaz N, Havel JJ, Makarov V, Desrichard A, Urba WJ, Sims JS, Hodi FS, Martin-Algarra S, Mandal R, Sharfman WH, et al: **Tumor and Microenvironment Evolution during Immunotherapy with Nivolumab.** *Cell* 2017.

5. Feng L, Qian H, Yu X, Liu K, Xiao T, Zhang C, Kuang M, Cheng S, Li X, Wan J, Zhang K: **Heterogeneity of tumor-infiltrating lymphocytes ascribed to local immune status rather than neoantigens by multi-omics analysis of glioblastoma multiforme.** *Sci Rep* 2017, **7:**6968.

6. Chen PL, Roh W, Reuben A, Cooper ZA, Spencer CN, Prieto PA, Miller JP, Bassett RL, Gopalakrishnan V, Wani K, et al: **Analysis of Immune Signatures in Longitudinal Tumor Samples Yields Insight into Biomarkers of Response and Mechanisms of Resistance to Immune Checkpoint Blockade.** *Cancer Discov* 2016, **6:**827-837.

7. Kirilovsky A, Marliot F, El Sissy C, Haicheur N, Galon J, Pages F: **Rational bases for the use of the Immunoscore in routine clinical settings as a prognostic and predictive biomarker in cancer patients.** *Int Immunol* 2016, **28:**373-382.

8. Tumeh PC, Harview CL, Yearley JH, Shintaku IP, Taylor EJ, Robert L, Chmielowski B, Spasic M, Henry G, Ciobanu V, et al: **PD-1 blockade induces responses by inhibiting adaptive immune resistance.** *Nature* 2014, **515:**568-571.

9. Anagnostou V, Smith KN, Forde PM, Niknafs N, Bhattacharya R, White J, Zhang T, Adleff V, Phallen J, Wali N, et al: **Evolution of Neoantigen Landscape during Immune Checkpoint Blockade in Non-Small Cell Lung Cancer.** *Cancer Discov* 2017, **7:**264-276.

10. Tulkens J, Lippens L, Vergauwen G, Jeurissen S, Dhondt B, Denys H, Hendrix A: **Extracellular vesicles to diagnose and treat cancer.** *Belgian Journal of Medical Oncology* 2017, **11:**92-105.

11. Thery C, Zitvogel L, Amigorena S: **Exosomes: composition, biogenesis and function.** *Nat Rev Immunol* 2002, **2:**569-579.

12. Colombo M, Raposo G, Thery C: **Biogenesis, secretion, and intercellular interactions of exosomes and other extracellular vesicles.** *Annu Rev Cell Dev Biol* 2014, **30:**255-289.

13. Hoshino A, Costa-Silva B, Shen TL, Rodrigues G, Hashimoto A, Tesic Mark M, Molina H, Kohsaka S, Di Giannatale A, Ceder S, et al: **Tumour exosome integrins determine organotropic metastasis.** *Nature* 2015, **527:**329-335.

14. Melo SA, Luecke LB, Kahlert C, Fernandez AF, Gammon ST, Kaye J, LeBleu VS, Mittendorf EA, Weitz J, Rahbari N, et al: **Glypican-1 identifies cancer exosomes and detects early pancreatic cancer.** *Nature* 2015, **523:**177-182.

15. Muller L, Muller-Haegele S, Mitsuhashi M, Gooding W, Okada H, Whiteside TL: **Exosomes isolated from plasma of glioma patients enrolled in a vaccination trial reflect antitumor immune activity and might predict survival.** *Oncoimmunology* 2015, **4:**e1008347.

16. Sandfeld-Paulsen B, Aggerholm-Pedersen N, Baek R, Jakobsen KR, Meldgaard P, Folkersen BH, Rasmussen TR, Varming K, Jorgensen MM, Sorensen BS: **Exosomal proteins as prognostic biomarkers in non-small cell lung cancer.** *Mol Oncol* 2016, **10:**1595-1602.

17. Del Re M, Marconcini R, Pasquini G, Rofi E, Vivaldi C, Bloise F, Restante G, Arrigoni E, Caparello C, Bianco MG, et al: **PD-L1 mRNA expression in plasma-derived exosomes is associated with response to anti-PD-1 antibodies in melanoma and NSCLC.** *Br J Cancer* 2018, **118:**820-824.

18. Valadi H, Ekstrom K, Bossios A, Sjostrand M, Lee JJ, Lotvall JO: **Exosome-mediated transfer of mRNAs and microRNAs is a novel mechanism of genetic exchange between cells.** *Nat Cell Biol* 2007, **9:**654-659.

19. Siravegna G, Marsoni S, Siena S, Bardelli A: **Integrating liquid biopsies into the management of cancer.** *Nat Rev Clin Oncol* 2017, **14:**531-548.

20. Lauber K, Ernst A, Orth M, Herrmann M, Belka C: **Dying cell clearance and its impact on the outcome of tumor radiotherapy.** *Front Oncol* 2012, **2:**116.

21. Herrera FG, Bourhis J, Coukos G: **Radiotherapy combination opportunities leveraging immunity for the next oncology practice.** *CA Cancer J Clin* 2017, **67:**65-85.

22. Demaria S, Golden EB, Formenti SC: **Role of Local Radiation Therapy in Cancer Immunotherapy.** *JAMA Oncol* 2015, **1:**1325-1332.

23. Vanpouille-Box C, Formenti SC, Demaria S: **TREX1 dictates the immune fate of irradiated cancer cells.** *Oncoimmunology* 2017, **6:**e1339857.

24. Diamond JM, Vanpouille-Box C, Spada S, Rudqvist NP, Chapman J, Ueberheide B, Pilones KA, Sarfraz Y, Formenti SC, Demaria S: **Exosomes shuttle TREX1-sensitive IFN-stimulatory dsDNA from irradiated cancer cells to dendritic cells.** *Cancer Immunol Res* 2018.

25. Gaipl US, Multhoff G, Scheithauer H, Lauber K, Hehlgans S, Frey B, Rodel F: **Kill and spread the word: stimulation of antitumor immune responses in the context of radiotherapy.** *Immunotherapy* 2014, **6:**597-610.

26. Vandenabeele P, Vandecasteele K, Bachert C, Krysko O, Krysko DV: **Immunogenic Apoptotic Cell Death and Anticancer Immunity.** *Adv Exp Med Biol* 2016, **930:**133-149.

27. Vanden Berghe T, Linkermann A, Jouan-Lanhouet S, Walczak H, Vandenabeele P: **Regulated necrosis: the expanding network of non-apoptotic cell death pathways.** *Nat Rev Mol Cell Biol* 2014, **15:**135-147.

28. Pasparakis M, Vandenabeele P: **Necroptosis and its role in inflammation.** *Nature* 2015, **517:**311-320.

29. Yu H, Guo P, Xie X, Wang Y, Chen G: **Ferroptosis, a new form of cell death, and its relationships with tumourous diseases.** *J Cell Mol Med* 2017, **21:**648-657.

30. Demaria S, Formenti SC: **Radiation as an immunological adjuvant: current evidence on dose and fractionation.** *Front Oncol* 2012, **2:**153.

31. Grice EA, Segre JA: **The human microbiome: our second genome.** *Annu Rev Genomics Hum Genet* 2012, **13:**151-170.

32. Sender R, Fuchs S, Milo R: **Are We Really Vastly Outnumbered? Revisiting the Ratio of Bacterial to Host Cells in Humans.** *Cell* 2016, **164:**337-340.

33. Odamaki T, Kato K, Sugahara H, Hashikura N, Takahashi S, Xiao JZ, Abe F, Osawa R: **Age-related changes in gut microbiota composition from newborn to centenarian: a cross-sectional study.** *BMC Microbiol* 2016, **16:**90.

34. Bonder MJ, Kurilshikov A, Tigchelaar EF, Mujagic Z, Imhann F, Vila AV, Deelen P, Vatanen T, Schirmer M, Smeekens SP, et al: **The effect of host genetics on the gut microbiome.** *Nat Genet* 2016, **48:**1407-1412.

35. Biedermann L, Zeitz J, Mwinyi J, Sutter-Minder E, Rehman A, Ott SJ, Steurer-Stey C, Frei A, Frei P, Scharl M, et al: **Smoking cessation induces profound changes in the composition of the intestinal microbiota in humans.** *PLoS One* 2013, **8:**e59260.

36. Zaura E, Brandt BW, Teixeira de Mattos MJ, Buijs MJ, Caspers MP, Rashid MU, Weintraub A, Nord CE, Savell A, Hu Y, et al: **Same Exposure but Two Radically Different Responses to Antibiotics: Resilience of the Salivary Microbiome versus Long-Term Microbial Shifts in Feces.** *MBio* 2015, **6:**e01693-01615.

37. Moschen AR, Wieser V, Tilg H: **Dietary Factors: Major Regulators of the Gut's Microbiota.** *Gut Liver* 2012, **6:**411-416.

38. Marchesi JR, Adams DH, Fava F, Hermes GD, Hirschfield GM, Hold G, Quraishi MN, Kinross J, Smidt H, Tuohy KM, et al: **The gut microbiota and host health: a new clinical frontier.** *Gut* 2016, **65:**330-339.

39. Jandhyala SM, Talukdar R, Subramanyam C, Vuyyuru H, Sasikala M, Nageshwar Reddy D: **Role of the normal gut microbiota.** *World J Gastroenterol* 2015, **21:**8787-8803.

40. Roy S, Trinchieri G: **Microbiota: a key orchestrator of cancer therapy.** *Nat Rev Cancer* 2017, **17:**271-285.

41. Garrett WS: **Cancer and the microbiota.** *Science* 2015, **348:**80-86.

42. Schwabe RF, Jobin C: **The microbiome and cancer.** *Nat Rev Cancer* 2013, **13:**800-812.

43. Fridman WH, Zitvogel L, Sautes-Fridman C, Kroemer G: **The immune contexture in cancer prognosis and treatment.** *Nat Rev Clin Oncol* 2017.

44. Viaud S, Saccheri F, Mignot G, Yamazaki T, Daillere R, Hannani D, Enot DP, Pfirschke C, Engblom C, Pittet MJ, et al: **The intestinal microbiota modulates the anticancer immune effects of cyclophosphamide.** *Science* 2013, **342:**971-976.

45. Iida N, Dzutsev A, Stewart CA, Smith L, Bouladoux N, Weingarten RA, Molina DA, Salcedo R, Back T, Cramer S, et al: **Commensal bacteria control cancer response to therapy by modulating the tumor microenvironment.** *Science* 2013, **342:**967-970.

46. Bronckaers A, Balzarini J, Liekens S: **The cytostatic activity of pyrimidine nucleosides is strongly modulated by Mycoplasma hyorhinis infection: Implications for cancer therapy.** *Biochem Pharmacol* 2008, **76:**188-197.

47. Lehouritis P, Cummins J, Stanton M, Murphy CT, McCarthy FO, Reid G, Urbaniak C, Byrne WL, Tangney M: **Local bacteria affect the efficacy of chemotherapeutic drugs.** *Sci Rep* 2015, **5:**14554.

48. Vande Voorde J, Sabuncuoglu S, Noppen S, Hofer A, Ranjbarian F, Fieuws S, Balzarini J, Liekens S: **Nucleoside-catabolizing enzymes in mycoplasma-infected tumor cell cultures compromise the cytostatic activity of the anticancer drug gemcitabine.** *J Biol Chem* 2014, **289:**13054-13065.

49. Guthrie L, Gupta S, Daily J, Kelly L: **Human microbiome signatures of differential colorectal cancer drug metabolism.** *NPJ Biofilms Microbiomes* 2017, **3:**27.

50. Nayak R, O'Loughlin C, Fischbach M, Turnbaugh P: **Methotrexate Is an Antibacterial Drug Metabolized By Human Gut Bacteria.** *Arthritis Rheumatol* 2016, **68 (suppl 10)**.

51. Alexander JL, Wilson ID, Teare J, Marchesi JR, Nicholson JK, Kinross JM: **Gut microbiota modulation of chemotherapy efficacy and toxicity.** *Nat Rev Gastroenterol Hepatol* 2017, **14:**356-365.

52. Nunes-Alves C: **Microbiome: Commensals promote anticancer immunotherapy.** *Nat Rev Micro* 2016, **14:**3-3.

53. Vetizou M, Pitt JM, Daillere R, Lepage P, Waldschmitt N, Flament C, Rusakiewicz S, Routy B, Roberti MP, Duong CP, et al: **Anticancer immunotherapy by CTLA-4 blockade relies on the gut microbiota.** *Science* 2015, **350:**1079-1084.

54. Sivan A, Corrales L, Hubert N, Williams JB, Aquino-Michaels K, Earley ZM, Benyamin FW, Lei YM, Jabri B, Alegre ML, et al: **Commensal Bifidobacterium promotes antitumor immunity and facilitates anti-PD-L1 efficacy.** *Science* 2015, **350:**1084-1089.

55. Gopalakrishnan V, Spencer CN, Nezi L, Reuben A, Andrews MC, Karpinets TV, Prieto PA, Vicente D, Hoffman K, Wei SC, et al: **Gut microbiome modulates response to anti-PD-1 immunotherapy in melanoma patients.** *Science* 2018, **359:**97-103.

56. Derosa L, Routy B, Enot D, Baciarello G, Massard C, Loriot Y, Fizazi K, Escudier BJ, Zitvogel L, Albiges L: **Impact of antibiotics on outcome in patients with metastatic renal cell carcinoma treated with immune checkpoint inhibitors.** *Journal of Clinical Oncology* 2017, **35**.

57. Ott PA, Bang YJ, Berton-Rigaud D, Elez E, Pishvaian MJ, Rugo HS, Puzanov I, Mehnert JM, Aung KL, Lopez J, et al: **Safety and Antitumor Activity of Pembrolizumab in Advanced Programmed Death Ligand 1-Positive Endometrial Cancer: Results From the KEYNOTE-028 Study.** *J Clin Oncol* 2017, **35:**2535-2541.

58. Schellens JHM, Marabelle A, Zeigenfuss S, Ding J, Pruitt SK, Chung HC: **Pembrolizumab for previously treated advanced cervical squamous cell cancer: Preliminary results from the phase 2 KEYNOTE-158 study.** *Journal of Clinical Oncology* 2017, **35:**5514-5514.

59. Smits LP, Bouter KE, de Vos WM, Borody TJ, Nieuwdorp M: **Therapeutic potential of fecal microbiota transplantation.** *Gastroenterology* 2013, **145:**946-953.

60. Forbes NS: **Engineering the perfect (bacterial) cancer therapy.** *Nat Rev Cancer* 2010, **10:**785-794.
